# Quantitative Proteomic Analysis in Alveolar Type II Cells Reveals the Different Capacities of RAS and TGF-β to Induce Epithelial–Mesenchymal Transition

**DOI:** 10.3389/fmolb.2021.595712

**Published:** 2021-03-19

**Authors:** Yilu Zhou, Charlotte Hill, Liudi Yao, Juanjuan Li, David Hancock, Julian Downward, Mark G. Jones, Donna E. Davies, Rob M. Ewing, Paul Skipp, Yihua Wang

**Affiliations:** ^1^ Biological Sciences, Faculty of Environmental and Life Sciences, University of Southampton, Southampton, United Kingdom; ^2^ Institute for Life Sciences, University of Southampton, Southampton, United Kingdom; ^3^ Oncogene Biology, The Francis Crick Institute, London, United Kingdom; ^4^ Clinical and Experimental Sciences, Faculty of Medicine, University of Southampton, Southampton, United Kingdom; ^5^ NIHR Southampton Biomedical Research Centre, University Hospital Southampton, Southampton, United Kingdom; ^6^ Centre for Proteomic Research, Institute for Life Sciences, University of Southampton, Southampton, United Kingdom

**Keywords:** epithelial-mesenchymal transition (EMT), RAS, TGF-β, lung disease, fibrosis, proteomics

## Abstract

Alveolar type II (ATII) epithelial cells function as stem cells, contributing to alveolar renewal, repair and cancer. Therefore, they are a highly relevant model for studying a number of lung diseases, including acute injury, fibrosis and cancer, in which signals transduced by RAS and transforming growth factor (TGF)-β play critical roles. To identify downstream molecular events following RAS and/or TGF-β activation, we performed proteomic analysis using a quantitative label-free approach (LC-HDMS^E^) to provide in-depth proteome coverage and estimates of protein concentration in absolute amounts. Data are available via ProteomeXchange with identifier PXD023720. We chose ATII^ER:KRASV12^ as an experimental cell line in which RAS is activated by adding 4-hydroxytamoxifen (4-OHT). Proteomic analysis of ATII cells treated with 4-OHT or TGF-β demonstrated that RAS activation induces an epithelial–mesenchymal transition (EMT) signature. In contrast, under the same conditions, activation of TGF-β signaling alone only induces a partial EMT. EMT is a dynamic and reversible biological process by which epithelial cells lose their cell polarity and down-regulate cadherin-mediated cell–cell adhesion to gain migratory properties, and is involved in embryonic development, wound healing, fibrosis and cancer metastasis. Thus, these results could help to focus research on the identification of processes that are potentially driving EMT-related human disease.

## 1 Introduction

Fibrosis and lung cancer present a large medical burden with lung cancer being the leading cause of death in the United States in both men and women (Torre et al., 2016). Fibrotic lung diseases, such as idiopathic pulmonary fibrosis (IPF), present a growing problem with limited treatments and poor prognosis (Richeldi et al., 2017; Hill et al., 2019a). Gas exchange in the lungs happens in alveolar sacs which are lined with two epithelial cell types (squamous alveolar type I, ATI and surfactant-secreting alveolar type II, ATII cells) (Pérez-Gil, 2008). It has been proposed that local signals are able to regulate ATII epithelial cells, which are able to function as stem cells contributing to alveolar renewal, repair and cancer (Desai et al., 2014). The integrity of these cell types is critical in the modulation of a number of diseases including cancer and fibrosis (Desai et al., 2018).

Certain signaling pathways have been demonstrated to be key in mediating a number of processes in lung-related disease. These signaling pathways have a diverse range of functions and subsequently affect a diverse range of biological processes. They are also known to interact with each other to influence their effects (Vallath et al., 2014; Aschner and Downey, 2016). Epidermal growth factor receptor (EGFR) is a transmembrane receptor tyrosine kinase activated by members of the EGF family (Linggi and Carpenter, 2006) and its pathway has been previously implicated in a number of lung pathologies, including cancer and fibrosis (Burgel and Nadel, 2008; Vallath et al., 2014; Yao et al., 2018). RAS is a downstream effector of EGFR and mutations in RAS have been widely reported in a number of cancers and other diseases (Downward, 2003; Tzouvelekis et al., 2013; Stella et al., 2014). RAS proteins switch between a GTP-bound “on-state” and a stable, GDP-bound “off-state”; this is mediated by G activating proteins (GAPs) and guanine nucleotide exchange factors (GEFs) which convert from active to inactive and inactive to active respectively (Simanshu et al., 2017). Once active, RAS stimulates a number of downstream signaling cascades which include mitogen-activated protein kinase (MAPK) and phosphatidylinositol-3 kinase (PI3K) (Downward, 2003). RAS signaling has been implicated in a number of cellular processes including proliferation, differentiation and apoptosis (Downward, 1998; Cox and Der, 2003; Sun et al., 2015).

Transforming growth factor (TGF)-β is a well-studied, multifunctional profibrotic cytokine known to play an important role in developmental biology (Shull et al., 1992; Kulkarni et al., 1993), cancer (Massagué, 2008), fibrotic disease (Leppäranta et al., 2012; Yao et al., 2018) and many other lung pathologies (Saito et al., 2018). Key mediators of the TGF-β family are Smads, which act together to regulate transcription (Massagué, 1998). In turn, these are involved in a number of cellular context-dependent processes including cell growth arrest (Mukherjee et al., 2010), proliferation (Carl et al., 2016; Cheng et al., 2016), apoptosis (Schuster and Krieglstein, 2002), and epithelial–mesenchymal transition (EMT) (Guo, 2017; Goldmann et al., 2018).

Our study utilized quantitative proteomic analysis to provide a comprehensive and unbiased investigation into the role of RAS and TGF-β signaling in ATII cells. In this study we identified EMT as a key pathway induced upon RAS-activation in ATII cells, but not in TGF-β-treated ATII cells in the given time. ATII cells with both RAS- and TGF-β signaling induced, leads to an EMT proteomic signature, which is mainly driven by RAS signaling. These findings are key to further understanding the mechanisms underlying lung disease.

## 2 Materials and Methods

### Cell Culture and Reagents

ATII^ER:KRASV12^ cells (Molina-Arcas et al., 2013; Coelho et al., 2017; Yao et al., 2018) were cultured in DCCM-1 (Biological Industries Ltd.) supplemented with 10% newborn calf serum (NBCS) (Life Technologies), 1% penicillin, 1% streptomycin and 1% l-glutamine (all from Sigma Aldrich). Cells were kept at 37°C and 5% CO_2_. To induce RAS activation in ATII^ER:KRASV12^ cells, 250 nM 4-OHT (Sigma-Aldrich) was added. TGF-β1 was from PeproTech. No *mycoplasma* contamination was detected in the cell lines used. For wet lab experiments, treatment with both 4-OHT (250 nM) and TGF-β1 (5 ng/ml) was for 24 h, unless otherwise indicated in the figure. For proteomic cell culture, ATII^ER:KRASV12^ cells were cultured as indicated above. Each treatment group (control, 4-OHT, TGF-β1 and combined (TGF-β1 and 4-OHT)) was conducted in triplicate and cells were treated for 24 h with indicated treatments prior to cell lysis.

### Western Blot Analysis

Western blot analysis was performed with lysates from cells lyzed with urea buffer (8 M Urea, 1 M Thiourea, 0.5% CHAPS, 50 mM DTT, and 24 mM Spermine). Primary antibodies were from: Santa Cruz (E-cadherin, sc-21791; ZEB1, sc-25388; Snail2, sc-10436), Abcam (β-tubulin, ab6046), Cell Signaling Technology (phospho-AKT, 9271; phospho-ERK, 9101; Snail1, 3879; Snail2, 9585; Phospho-Smad2, 3104; *β*-tubulin, 86298), and BD Transduction Laboratories (E-cadherin, 610405). Signals were detected using an Odyssey imaging system (LI-COR), and evaluated by ImageJ 1.42q software (National Institutes of Health).

### qRT-PCR

Total RNA was isolated using RNeasy mini kit (Qiagen) according to the manufacturer’s instructions and quantified using a Nanodrop Spectrophotometer 2000c (Thermo Fisher Scientific). Real-time quantitative RT-PCR was carried out using gene-specific primers (QuantiTect Primer Assays, Qiagen) for *CDH1* (E-cadherin) (QT00080143), *SNAI1* (Snail1) (QT00010010), *SNAI2* (Snail2) (QT00044128), *ZEB1* (QT00008555), *ZEB2* (QT00008554), *TWIST* (QT00011956), *VIM* (QT00095795), *TGFBR1* (QT00083412), *TGFBR2* (QT00014350), *TGFBR3* (QT00083223) or *ACTB* (β-actin) (QT01680476) with QuantiNova SYBR Green RT-PCR kits (Qiagen). Relative transcript levels of target genes were normalized to *ACTB* (β-actin).

### Proteomics Sample Preparation

Samples were lyzed in 0.1 M ammonium bicarbonate containing 1% SDS followed by pulsed sonication. Lysates were then centrifuged at 13,000 x g, for 20 min at 4°C, supernatant removed and protein concentration measured using a DirectDetect spectrometer (Merck, United Kingdom).

Methanol/chloroform extraction was then performed on 25 μg of protein for each lysate. Samples were prepared in 100 μL using lysis buffer (0.1 M ammonium bicarbonate). Samples were resuspended in 100 μl of 0.1 M ammonium bicarbonate containing 0.1% SDS and reduced with 1 mM Dithiothreitol (DTT, Thermo Scientific) at 56°C for 60 min. Samples were subsequently alkylated using 5.5 mM iodoacetamide (Sigma) and incubated in the dark at room temperature for 45 min. Samples were digested by addition of 2 μg sequencing grade modified trypsin (Promega) and incubated overnight at 37°C. After digestion, samples were lyophilized in vacuo.

Enolase protein digest internal standard (Waters) was spiked into each cellular peptide sample at a concentration of 150 fmol prior to isoelectric focusing by OFFGEL fractionation using a 3–10 pH gradient (Agilent) into 12 peptide fractions. Each fraction was purified using an Empore™ C18 solid phase extraction plate (ThermoScientific Pierce) to remove residual salts, buffers and contaminants before lyophilization and resuspension in loading buffer (3% acetonitrile + 0.1% formic acid) for mass spectrometry analysis.

Separations were performed using a nanoAcquity UPLC system (Waters). Peptide digests were injected onto a Symmetry C18, 180 µm × 20 mm trapping cartridge (Waters). After 5 min washing of the trap column, peptides were separated using a 75 µm i. d. x 500 mm, 1.7 µm BEH130 C18, column (Waters) using a linear gradient of 5–40% B (buffer A = 0.1% formic acid in water, buffer B = 0.1% formic acid in acetonitrile) over 90 min with a wash to 85% B at a flow rate of 300 nL/min. All separations were automated, performed on-line and sprayed directly into the nanospray source of the mass spectrometer.

All mass spectrometry was performed using a Waters G2-Si Synapt HDMS mass spectrometer operating in MS^e^ mode. Data was acquired from 50 to 2000 *m/z* with ion mobility enabled using alternate low and high collision energy (CE) scans. Low CE was 5 V and elevated, ramped from 20–40 V. The lock mass Glu-fibrinopeptide (M+2H)^+2^, *m/z* = 785.8426) was infused at 300 nl/min at a concentration of 200 fmol/μL and acquired every 13 s.

Raw mass spectra were processed using ProteinLynx Global Server Ver 3.0 (Waters, Manchester, United Kingdom) enabled using an in-house developed script and the data processed to generate reduced charge state and de-isotoped precursor and associated product ion mass lists. These peak lists were searched against the human UniProt protein sequence (downloaded March 2017). A maximum of one missed cleavage was allowed for tryptic digestion and the variable modification was set to contain oxidation of methionine and carboxyamidomethylation of cysteine was set as a fixed modification. The false discovery rate (FDR) was estimated with decoy-fusion database searches and were filtered to 1% FDR.

### Proteomic Sample Normalization and Imputation

Raw data was imported into RStudio software (v. 1.1.456) for down-stream analysis. Expression values of proteins in femtomole (fmol) were extracted and normalized using the variance stabilization normalization (Vsn) method (Valikangas et al., 2018a). The Vsn method was implemented using the justvsn function of the Vsn package in RStudio. This method was utilized as a recent review of normalization methods for use in quantitative label-free proteomics determined that it reduced variation the most between technical replicates compared to other tested methods (Huber et al., 2002). Normalized data were transformed into log2 format. Missing values were imputed using local least squares imputation (Lls) with parameter value 150 (Valikangas et al., 2018b), using pcaMethods package (Stacklies et al., 2007).

Proteins had to be observed in at least two of three replicates to be used in the experiment. Samples were normalized and missing values imputed, before PCA (Supplementary Figure. S3) validated samples clustered appropriately, differentially expressed proteins were analyzed by hierarchal clustering and visualized on a heatmap (Supplementary Figures S1B, S2B).

The limma package (v. 3_34.2) was utilized to detect differentially expressed proteins (DEPs) (Ritchie et al., 2015). DEPs were determined by *p* Value <0.05 and ∣log_2_ [Fold Change]∣ ≥0.5. All expression data after limma is available in Supplementary Tables S1, S3, S5. These are the top table export from limma, further details about these can be found in the limma user guide (Ritchie et al., 2015). The mass spectrometry proteomics data have been deposited to the ProteomeXchange Consortium via the PRIDE (Perez-Riverol et al., 2019) partner repository with the dataset identifier PXD023720.

### Pathway Analysis

Prior to pathway analysis protein symbols were converted to gene symbols in RStudio (v. 3.6.1). Enrichment analyses were generated by Metascape (Zhou et al., 2019), targets were analyzed with hallmark annotation.

### Statistical Analysis and Repeatability of Experiments

Proteomic experiments were performed in triplicate. Unless otherwise noted, data are presented as mean and s.d., and a two-tailed, unpaired or paired Student's *t*-test was used to compare two groups for independent samples, with the n number for each experiment included in the figure legends. *p* < 0.05 was considered statistically significant.

## 3 Results

### RAS-Activation in Alveolar Type II Cells Induces Epithelial–Mesenchymal Transition

To characterize the proteome-wide altered expression in ATII cells, we performed quantitative mass spectrometry. We utilized a RAS-inducible model in ATII^ER:KRASV12^ cells (Molina-Arcas et al., 2013; Coelho et al., 2017; Yao et al., 2018). KRASV12 (containing a single amino acid mutation in KRAS, glycine to valine at position 12) fused to the estrogen receptor (ER) ligand-binding domain was introduced into ATII cells to generate ATII^ER:KRASV12^, where the RAS pathway is activated by the addition of 4-hydroxytamoxifen (4-OHT). Mass spectrometry identified 1844 proteins, of these 182 were determined to be differentially expressed proteins (DEPs) (*p* Value <0.05, ∣log_2_ (fold change [FC])∣ ≥ 0.5) (Supplementary Table S1).

**FIGURE 1:**
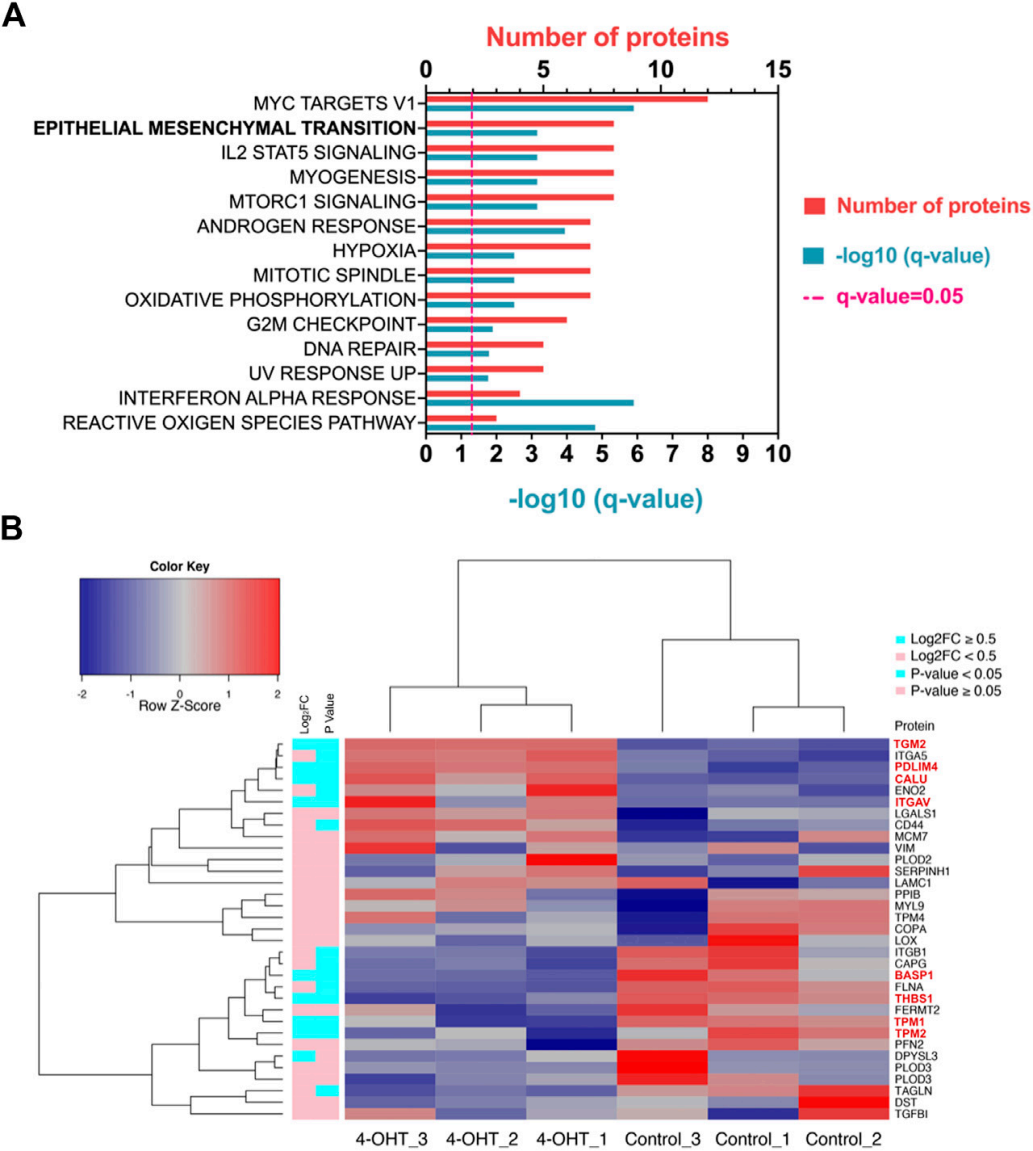
RAS-activation in ATII cells induces Hallmark EMT proteomic signature **(A)** Pathway analysis in Metascape (Metascape.com) analyzing differentially expressed proteins (DEPs) in ATII^ER:KRASV12^ cells treated with 250 nM 4-OHT or control for 24 h, all non-significant pathways have been removed from diagram. **(B)** Heatmap and hierarchical cluster analysis of ATII^ER:KRASV12^ cells treated with 250 nM 4-OHT (RAS-activated) compared with control for 24 h, a panel of hallmark EMT proteins were evaluated. *p* Value (blue: *p* Value <0.05, pink: *p* Value ≥ 0.05). ∣log_2_ fold change (blue: ∣log_2_FC∣ ≥ 0.5, pink: ∣log_2_FC∣ < 0.5). Red text indicates differentially expressed proteins (DEPs).

RAS signaling is important in a number of different processes. In order to determine which pathways are altered when ATII cells undergo RAS activation, pathway analysis was conducted (Figure 1A). Hallmark epithelial-mesenchymal transition (EMT) was identified as one of the key pathways altered upon RAS activation in ATII cells (Figure 1A, *q* Value = 0.0007, shared genes = 8). A full list of altered pathways is included in Table 1, including shared genes identified in the analysis.

**TABLE 1:** Hallmark pathways identified using Metascape (Metascape.com) for differentially expressed proteins between control and RAS-activated ATII^ER:KRASV12^ cells.

Hallmark pathways	Log (*q* Value)	Shared genes	Number of shared genes	UniProt ID
Hallmark myc targets v1	−5.900	CANX, EEF1B2, ETF1, XRCC6, KPNB1, MCM5, NAP1L1, PGK1, SYNCRIP, RUVBL2, EXOSC7, LSM2	12/200	P27824, P24534, P62495, P12956, Q14974, P33992, P55209, P00558, O60506, Q9Y230, Q15024, Q9Y333
Hallmark IL2 STAT5 signaling	−3.160	ENO3, ITGA6, ITGAV, PNP, PLEC, TGM2, NDRG1, AKAP2	8/200	P13929, P23229, P06756, P00491, Q15149, P21980, Q92597, Q9Y2D5
Hallmark epithelial- mesenchymal transition	−3.160	CALU, ITGAV, TGM2, THBS1, TPM1, TPM2, PDLIM4, BASP1	8/200	O43852, P06756, P21980, P07996,P09493, P07951, P50479, P80723
Hallmark myogenesis	−3.160	ACTN3, DES, NQO1, ENO3, MYH2, MYH9, TPM2, TPM3	8/200	Q08043, P17661, P15559, P13929, Q9UKX2, P35579, P07951, P06753
Hallmark MTORC1 signaling	−3.160	CANX, DHCR7, ETF1, PNP, PGK1, PSMB5, RRM2, STIP1	8/200	P27824, Q9UBM7, P62495, P00491, P00558, P28074,P31350, P31948
Hallmark androgen response	−3.949	ACTN1, XRCC6, ITGAV, TPD52, AKAP12, NDRG1, ADRM1	7/101	P12814,P12956,P06756,P55327,Q02952,Q92597,Q16186
Hallmark hypoxia	−2.510	ENO3, MYH9, PGK1, TGM2, TPD52, AKAP12, NDRG1	7/200	P13929,P35579,P00558,P21980,P55327,Q02952,Q92597
Hallmark mitotic spindle	−2.510	FLNB, MARCKS, MYH9, NUMA1, CLIP1, FSCN1, ANLN	7/200	O75369, P29966, P35579, Q14980, P30622, Q16658, Q9NQW6
Hallmark oxidative phosphorylation	−2.510	DLST, ETFB, IDH3A, NDUFV2, PMPCA, TOMM22, GRPEL1	7/200	P36957, P38117, P50213, P19404, Q10713, Q9NS69, Q9HAV7
Hallmark G2M checkpoint	−1.897	DR1, KPNB1, MARCKS, MCM5, NUMA1, SYNCRIP	6/200	Q01658, Q14974, P29966, P33992, Q14980, O60506
Hallmark DNA repair	−1.791	PNP, POLR2H, EDF1, ADRM1, AK3	5/150	P00491, P52434, O60869, Q16186, Q9UIJ7
Hallmark UV response up	−1.766	CDC5L, DNAJA1, POLR2H, STIP1, GRPEL1	5/158	Q99459, P31689, P52434, P31948, Q9HAV7
Hallmark interferon alpha response	−5.900	ADAR, CD47, TRIM25, IFITM2	4/97	P55265, Q08722, Q14258, Q01629
Hallmark reactive oxygen species pathway	−4.809	NQO1, TXN, OXSR1	3/49	P15559, P10599, O95747

RAS-activation has previously been demonstrated to be instrumental in the induction of EMT (Wang et al., 2010; Arner et al., 2019). To evaluate the role of RAS-activation in ATII cells on EMT processes, a selection of previously described Hallmark EMT proteins (Subramanian et al., 2005) were investigated in our proteomic dataset. Of the identified 1844 proteins, 33 identified were EMT-related proteins, as assessed by the Hallmark EMT list. Of the 33 EMT proteins found, 8 were differentially expressed between control and RAS-activated, as indicated by red text (*p* Value <0.05, ∣log_2_FC∣ ≥ 0.5) (Figure 1B). All 33 EMT-proteins identified in the proteomics data were visualized on a heatmap, a clear separation is observed between RAS-activated (4-OHT) and control (Figure 1B), suggesting a clear role for EMT in RAS-activated ATII cells. Further, all samples have been visualized on a volcano plot (Supplementary Figure S1A) and a heat map showing DEPs (Supplementary Figure S1B). Significantly up- and down-regulated EMT-proteins have been identified (Supplementary Figure S1A; Supplementary Table S2).

### Transforming Growth Factor-β Does Not Induce a Hallmark Epithelial–Mesenchymal Transition in Proteomics Pathway Analysis

Given the extensive role of TGF-β in numerous lung pathologies, determining which pathways are altered in alveolar epithelial cells could be key in elucidating disease pathology. Proteomic analysis was also conducted on the ATII cells treated with and without TGF-β (5 ng/ml for 24 h). Mass spectrometry identified 1858 proteins, of which 90 were DEPs (*p* Value <0.05, ∣log_2_FC∣ ≥ 0.5) (Supplementary Table S3).

**FIGURE 2:**
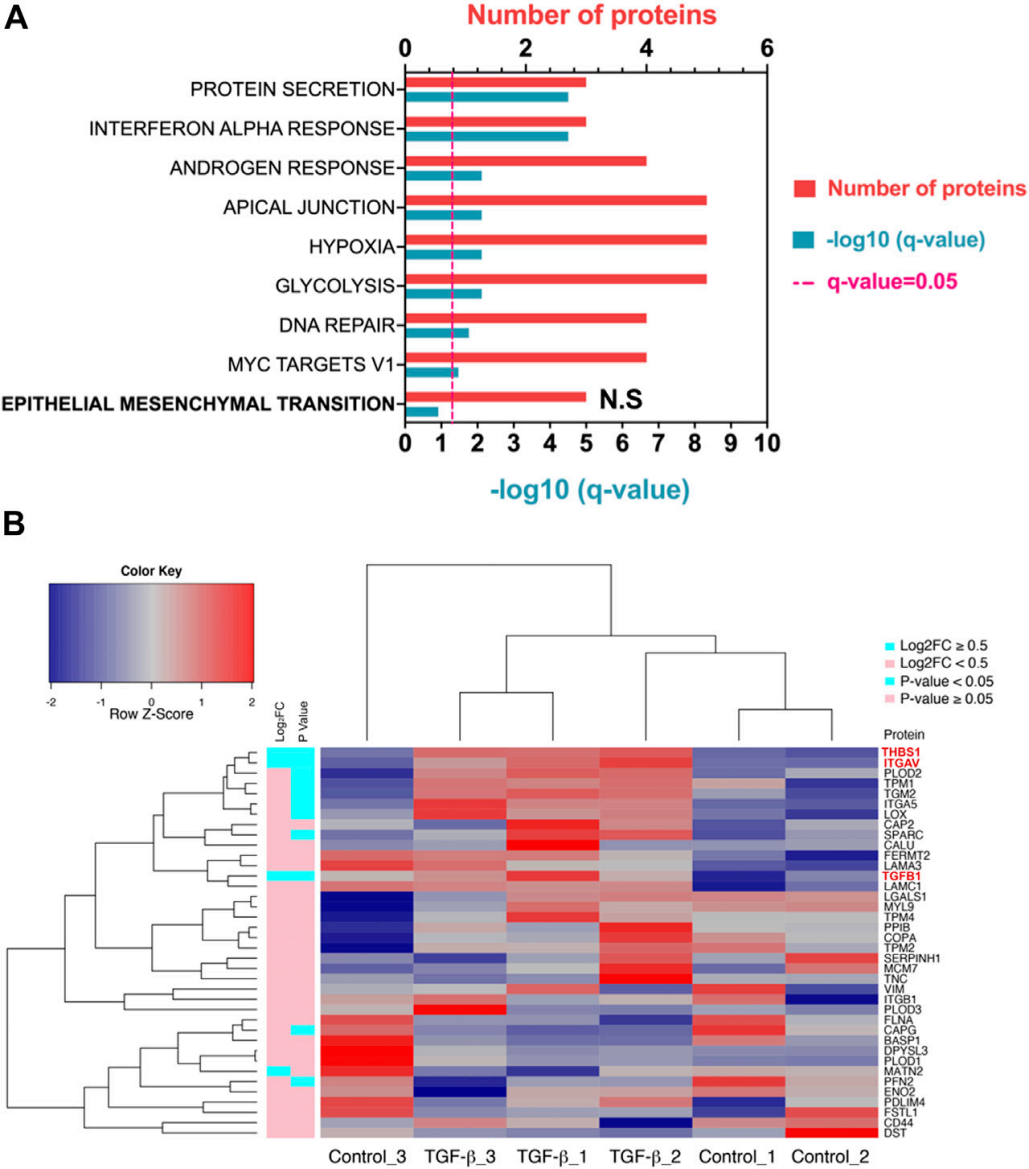
TGF-β in ATII cells does not induce a classical EMT program at a given time point. **(A)** Pathway analysis in Metascape (Metascape.com) analyzing differentially expressed proteins (DEPs) in ATII^ER:KRASV12^ cells treated with 5 ng/ml TGF-β or control for 24 h, all non-significant pathways have been removed from the diagram with the exception of epithelial-mesenchymal transition (EMT) which was not significant and has been highlighted in bold. **(B)** Heatmap and hierarchical cluster analysis of ATII^ER:KRASV12^ cells treated with 5 ng/ml TGF-β or control treated for 24 h, a panel of hallmark EMT proteins were evaluated. *p* Value (blue: *p* Value <0.05, pink: *p* Value ≥ 0.05). Absolute log_2_ fold change (blue: ∣log_2_FC∣ ≥ 0.5, pink: ∣log_2_FC∣ < 0.5). Red text indicates differentially expressed proteins (DEPs).

Downstream pathway analysis of TGF-β treated ATII cells did not indicate Hallmark EMT to be significantly induced (Figure 2A, *q* Value = 0.12). All significantly altered pathways have been indicated, in addition to Hallmark EMT. A full list of altered pathways is included in Table 2, including shared genes found in the analysis. Hallmark EMT processes did share three common proteins with the dataset, but these were not significantly altered.

**TABLE 2:** Hallmark pathways identified using Metascape (Metascape.com) for differentially expressed proteins between control and TGF-β-treated ATII^ER:KRASV12^ cells.

Hallmark pathway	Log (*q* Value)	Shared genes	Number of shared genes	UniProt ID
Hallmark protein secretion	−4.507	KRT18, TPD52, STX7	3/96	P05783, P55327, O15400
Hallmark interferon alpha response	−4.507	CD47, TRIM25, IFITM2	3/97	Q08722, Q14258, Q01629
Hallmark androgen response	−2.115	ACTN1, ITGAV, TPD52, RRP12	4/101	P12814, P06756, P55327, Q5JTH9
Hallmark hypoxia	−2.115	ENO3, MYH9, PGK1, TGFBI,T PD52	5/200	P13929, P35579, P00558, Q15582, P55327
Hallmark apical junction	−2.115	ACTB, ACTN1, DLG1, MYH9, TGFBI	5/200	P60709, P12814, Q12959, P35579, Q15582
Hallmark glycolysis	−2.115	PGK1, PKM, PYGB, TGFBI, TXN	5/200	P00558, P14618, P11216, Q15582, P10599
Hallmark DNA repair	−1.763	POLR2H, RALA, ALYREF, SF3A3	4/150	P52434, P11233, Q86V81, Q12874
Hallmark myc targets v1	−1.475	EEF1B2, KPNB1, PGK1, SYNCRIP	4/150	P24534, Q14974, P00558, O60506
Hallmark epithelial-mesenchymal transition	−0.916	THBS1,ITGAV, TGFBI	3/200	P07996, P06756, Q15582

The same group of Hallmark EMT proteins, as previously described, were compared to the identified proteins and 38 EMT proteins were identified, three were differentially expressed (*p* Value <0.05, ∣log_2_FC∣ ≥0.5) (all were up-regulated) (Supplementary Table S4). A heat map illustrates all Hallmark EMT proteins which were identified in the proteomic dataset based on hierarchal clustering DEPs are highlighted by red text (Figure 2B). Clustering TGF-β samples based on Hallmark EMT proteins did not cause defined separation, with Control_3 clustering with TGF-β samples. Further, there was not a defined split in up- and down-regulated EMT proteins. A volcano of all differentially expressed proteins is shown in Supplementary Figure S2A, where the three EMT proteins which were significantly altered are labeled (THBS1, ITGAV and TGFB1). Further a heat map shows all DEPs upon TGF-β treatment (Supplementary Figure S2B).

### RAS-Activation Dominates Over Transforming Growth Factor-β in Driving Epithelial–Mesenchymal Transition in Alveolar Type II Cells

Given the different EMT response to RAS and TGF-β signaling activation we wanted to determine the effect of dual treatment and to elucidate if one pathway dominated in EMT-induction. In ATII cells with RAS and TGF-β signaling active, mass spectrometry identified 1793 proteins, of which 123 were differentially expressed between control and treatment (*p* Value <0.05, ∣log_2_FC∣ ≥ 0.5) (Supplementary Table S5).

**FIGURE 3:**
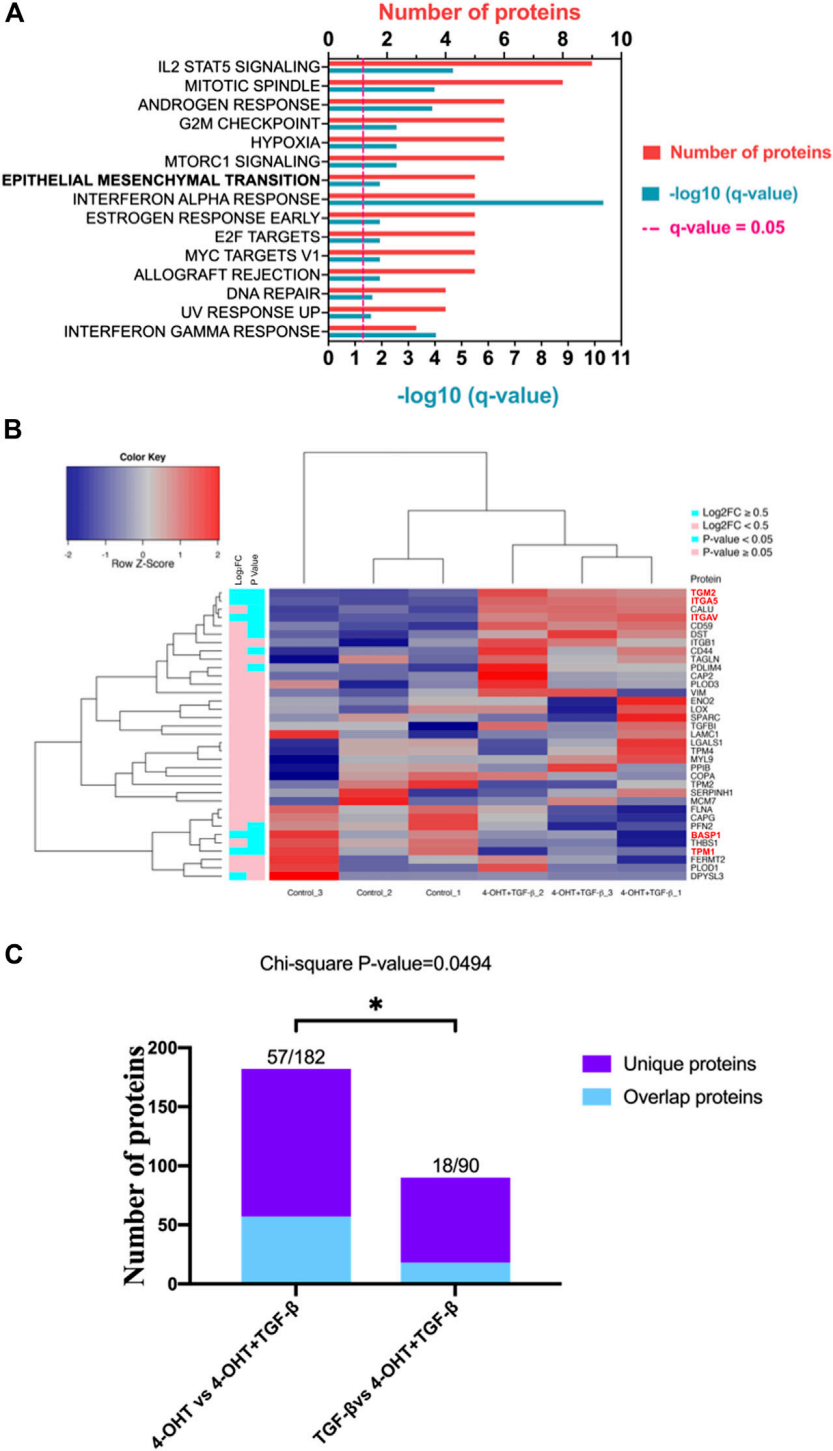
RAS-activation together with TGF-β are capable of inducing EMT and RAS signaling appears to drive EMT. **(A)** Pathway analysis in Metascape (Metascape.com) analyzing differentially expressed proteins (DEPs) in ATII^ER:KRASV12^ cells treated with a combined treatment of 250 nM 4-OHT and 5 ng/ml TGF-β or control for 24 h, all non-significant pathways have been removed from the diagram. **(B)** Heatmap and hierarchical cluster analysis of ATII^ER:KRASV12^ cells treated with a combined treatment of 250 nM 4-OHT and 5 ng/ml TGF-β or control for 24 h, a panel of hallmark EMT proteins were evaluated. *p* Value (blue: *p* Value < 0.05, pink: *p* Value ≥ 0.05). Absolute log_2_ fold change (blue: ∣log_2_FC∣ ≥ 0.5, pink: ∣log_2_FC∣ < 0.5). Red text indicates differentially expressed proteins (DEPs). **(C)** Diverging bar chart indicating the number of uniquely differentially expressed proteins in indicated treatments (Chi-squared test, *p* Value = 0.0494).

We previously observed Hallmark EMT to be a significant pathway for RAS-activated (Figure 1A, *q* Value = 0.0005) but not in TGF-β treatment alone (Figure 2A, *q* Value = 0.12), hence we wanted to evaluate the effect of RAS-activation together with TGF-β treatment on ATII^ER:KRASV12^ cells. The same group of Hallmark EMT proteins, as previously described, were compared to identified proteins and 35 EMT proteins were identified, five were differentially expressed (*p* Value <0.05, ∣log_2_FC∣ ≥ 0.5) (Supplementary Table S6). Pathway analysis identified Hallmark EMT (Figure 3A, *q* Value = 0.01) with five shared proteins. A full list of altered pathways is included in Table 3, including shared genes found in the analysis. When all Hallmark EMT proteins are visualized using a heat map based on hierarchal clustering, a more defined separation is observed, where DEPs are signified by red text (Figure 3B), compared with TGF-β treatment alone (Figure 2B). Dendrograms show a clear divide between control and RAS-activated/TGF-β treatment, however Control_3 appears to cluster separately from other control samples. We previously confirmed a global view of sample uniformity by PCA (Supplementary Figure S3). These results suggest that hallmark EMT is also induced in RAS-activated/TGF-β treated ATII^ER:KRASV12^ cells.

**TABLE 3:** Hallmark pathways identified using Metascape (Metascape.com) for differentially expressed proteins between control and TGF-β-treated RAS-activated ATII^ER:KRASV12^ cells.

Hallmark pathways	Log (*q* Value)	Shared genes	Number of shared genes	UNIPROT ID
Hallmark IL2 STAT5 signaling	−4.682	ENO3, ITGA6, ITGAV, PNP, PLEC, TGM2, GSTO1, NDRG1, AKAP2	9/200	P13929, P23229, P06756, P00491, Q15149, P21980, P78417, Q92597, Q9Y2D5
Hallmark mitotic spindle	−3.986	FLNB, KIF11, LMNB1, MARCKS, NUMA1, FSCN1, SPTBN1, NDC80	8/200	O75369, P52732, P20700, P29966, Q14980, Q16658, Q01082, O14777
Hallmark androgen response	−3.900	CDK6, XRCC6, H1F0, ITGAV, AKAP12, NDRG1	6/101	Q00534, P12956, P07305, P06756, Q02952, Q92597
Hallmark G2M checkpoint	−2.564	KIF11, LMNB1, MARCKS, NUMA1, NDC80, TACC3	6/200	P52732, P20700, P29966, Q14980, O14777, Q9Y6A5
Hallmark hypoxia	−2.564	ENO3, PFKP, TGM2, AKAP12, NDRG1, CAVIN1	6/200	P13929, Q01813, P21980, Q02952, Q92597,Q6NZI2
Hallmark MTORC1 signaling	−2.564	Canx, IDH1, PNP, PSMD12, RAB1A, RRM2	6/200	P27824, O75874, P00491, O00232, P62820, P31350
Hallmark interferon alpha response	−10.327	CD47, IFI35, IFIT3, IFITM2, NUB1	5/97	Q08722, P80217, O14879, Q01629, Q9Y5A7
Hallmark epithelial- mesenchymal transition	−1.932	ITGA5, ITGAV, TGM2, TPM1, BASP1	5/200	P08648, P06756, P21980, P09493, P80723
Hallmark estrogen response early	−1.932	Flnb, KRT15,PODXL, TGM2, MYOF	5/200	O75369, P19012, O00592, P21980, Q9NZM1
Hallmark E2F targets	−1.932	XRCC6, LMNB1, NME1, RRM2, TACC3	5/200	P12956, P20700, P15531, P31350, Q9Y6A5
Hallmark myc targets v1	−1.932	CANX, EEF1B2, XRCC6, NME1, NPM1	5/200	P27824, P24534, P12956, P15531, P06748
Hallmark allograft rejection	−1.932	CD47, EIF5A, HLA-A, NME1, NPM1	5/200	Q08722, P63241, P01891, P15531, P06748
Hallmark DNA repair	−1.652	NME1, PNP, POLR2H, RALA	4/150	P15531, P00491, P52434, P11233
Hallmark UV response up	−1.601	DNAJA1, POLR2H, RRAD, GRPEL1	4/158	P31689, P52434, P55042, Q9HAV7
Hallmark interferon gamma response	−4.036	IFI35, IFIT3, IFITM2	3/200	P80217, O14879, Q01629

Quantitative proteomic analysis identified different signatures of EMT in each treatment (RAS-activated, TGF-β treatment alone and combined RAS activation with TGF-β treatment) and we wanted to determine if a certain signaling pathway dominated. A chi-squared test (Figure 3C) demonstrated that when comparing A and B (A:RAS-activation vs RAS- activation with TGF-β, or B: TGF-β vs RAS-activation with TGF-β), there are a significant number of uniquely differentially expressed proteins (*p* Value = 0.0494). This analysis suggested that RAS-activation (4-OHT) dominates in the combined treatment of ATII^ER:KRASV12^ cells.

### RAS-Activation, but not Transforming Growth Factor-β, Results in Reduction in E-Cadherin in Alveolar Type II Cells

**FIGURE 4:**
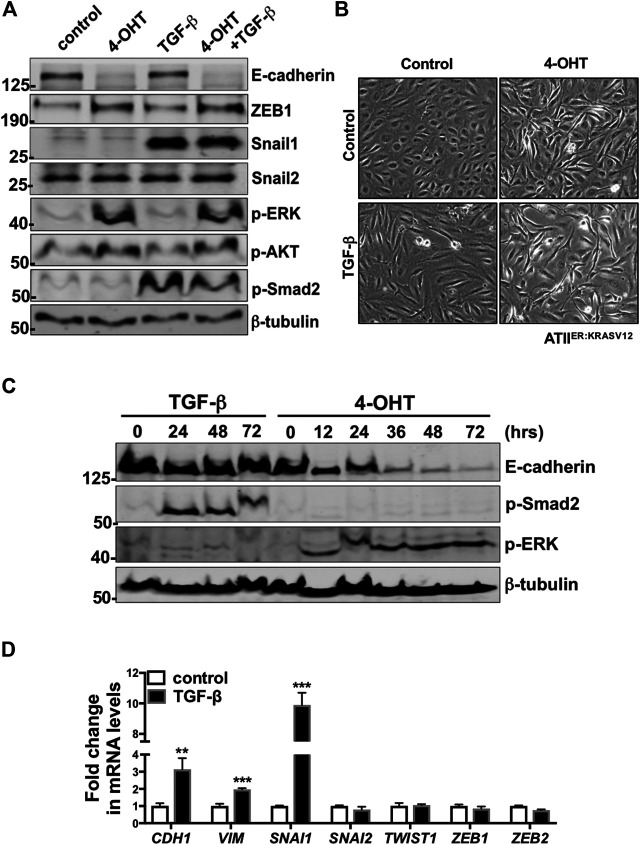
Unlike RAS-activation, TGF-β is insufficient to induce EMT-like changes at 24 h in ATII^ER:KRASV12^ cells. **(A)** Protein expression of E-cadherin, ZEB1, Snail1, Snail2, *p*-ERK, *p*-AKT, *p*-Smad2 in ATII^ER:KRASV12^ cells with indicated treatments at 24 h, *β*-tubulin was used as a loading control. *n* = 2 **(B)** Phase contrast images of ATII^ER:KRASV12^ cells with indicated treatment for 24 h. **(C)** Protein expression of E-cadherin, *p*-ERK, *p*-Smad2 in ATII^ER:KRASV12^ cells with 250 nM 4-OHT or 5 ng/ml TGF-β at indicated time points, *β*-tubulin was used as a loading control. *n* = 2 **(D)** Fold change in the mRNA level of *CDH1* (E-cadherin), *VIM* (Vimentin), *SNAI1*, *SNAI2*, *TWIST*, *ZEB1*, *ZEB2* in ATII^ER:KRASV12^ cells with indicated treatment for 24 h. *ACTB* (β-actin)-normalized mRNA levels in control cells were used to set the baseline value at unity. Data are mean ± s.d. *n* = 3 samples per group. **p < 0.01. ***p < 0.001.

We first demonstrated the presence of TGF-β receptor 1 (*TGFBR1*) and 2 (*TGFBR2*) in ATII cells (Supplementary Figure S4). In addition, phospho-Smad2 (*p*-Smad2) levels were increased upon TGF-β treatment (Figure 4A). These results suggested intact TGF-β signaling in these cells. To confirm the findings from our proteomic analysis, which identified induction of Hallmark EMT in RAS-activated samples but not in TGF-β treatment alone, we sought to validate protein levels of a number of EMT markers by western blot (Figure 4A). At the time points utilized (t = 24 h) protein levels of E-cadherin, a key cell-cell adhesion molecule and an epithelial marker, was significantly reduced upon RAS-activation and RAS-activation together with TGF-β treatment. However, where ATII^ER:KRASV12^ cells were treated with just TGF-β, a reduction in E-cadherin was not observed. The levels of tight junction proteins ZO1 and ZO2 were also investigated in our proteomic dataset (Supplementary Figure S5). No significant differences were observed in ZO1, however ZO2 was significantly reduced upon RAS-activation and TGF-β treatment. We then examined the levels of a number of EMT-Transcription Factors (EMT-TF) altered in all treatments; as previously described (Yao et al., 2018) upon RAS-activation ZEB1 was increased, TGF-β treatment led to up-regulation of Snail1 and dual activation of by RAS and TGF-β treatment led to up-regulation of both ZEB1 and Snail1. Further, after 24 h, phase contrast images of RAS-activated ATII^ER:KRASV12^ cells show the change to a spindle-like morphology from the cuboidal morphology of controls, within the same time frame less pronounced changes are observed by TGF-β treatment (Figure 4B).

Having examined the effect of each treatment at one time point (t = 24 h), we next evaluated whether alterations in E-cadherin protein levels were time-dependent. A time course of RAS-activated (4-OHT) and TGF-β treated ATII^ER:KRASV12^ cells determined protein levels of E-cadherin at a number of times points up to 72 h (Figure 4C). E-cadherin protein levels remain unaltered with TGF-β treatment at all time points examined, however reduction in E-cadherin is evident in RAS-activated ATII^ER:KRASV12^ cells from 12 h, with a large reduction observed at 36 h. To further elucidate the potential role of TGF-β in EMT in ATII^ER:KRASV12^ cells, mRNA levels of a number of EMT markers were evaluated (Figure 4D). Interestingly, levels of *VIM* (Vimentin), a structural protein mainly expressed in mesenchymal cells, were increased with TGF-β*.* While *CDH1* (E-cadherin) levels increased upon TGF-β treatment. We evaluated a number of EMT-TFs and of these only *SNAI1* (Snail1) was increased. *SNAI1* is important in the direct repression of E-cadherin, however levels of *CDH1* (E-cadherin) and protein levels of E-cadherin are not down-regulated, suggesting *SNAI1* may mediate some form of partial EMT in these cells.

## 4 Discussion

ATII epithelial cells are able to function as stem cells and subsequently contribute to a number of processes such as renewal, repair and the development of cancer (Desai et al., 2014). A number of key signaling pathways have been implicated in lung disease and to better understand their roles we performed quantitative label-free approach (LC-HDMS^E^), to compare the capacity of these signaling pathways. ATII cells with RAS activated and/or TGF-β treatment were utilized and hallmark pathways identified. RAS-activation alone was sufficient to induce an EMT signature. In comparison, activation of TGF-β signaling was insufficient to induce an EMT signature in the time period. When both RAS- and TGF-β- signaling were activated an EMT signature was induced and this was driven by RAS-signaling.

EMT is a well-established concept, it requires the involvement of a number of signaling pathways (Thiery and Sleeman, 2006) and the induction of EMT varies significantly in different tissues and diseases (Kalluri and Weinberg, 2009). As described, a number of signaling factors are involved in the induction of EMT, including TGF-β, fibroblast growth factor (FGF), Wnt/β-catenin and EGF. In turn, these can regulate the expression of a group of EMT specific transcription factors termed EMT-TF which are capable of orchestrating the induction of EMT, these include Snail1/2, ZEB1/2 and some basic helix-loop-helix (bHLH) factors. EMT-TFs have been demonstrated to be potent repressors of E-cadherin (Peinado et al., 2007; Nieto, 2011), which is considered to be a key step in the induction of EMT (Wheelock et al., 2008; Lamouille et al., 2014).

Previous studies have begun to compare the signaling mechanisms which drive EMT in malignancy (Taube et al., 2010), here we examine the mechanisms which drive EMT in the lung. EMT has roles in developmental biology, cancer and fibrosis and wound healing (Thiery et al., 2009; Barriere et al., 2015). In cancer, EMT has been demonstrated as a mechanism which allows cancer cells to metastasize to secondary tumor sites (Li and Balazsi, 2018). In fibrosis, the role of EMT is controversial. IPF is a chronic, interstitial lung disease which lacks both effective treatment and a clear understanding of underlying disease mechanisms (Richeldi et al., 2017; Hill et al., 2019a). A number of studies have proposed that fibroblasts are derived from epithelial cells by EMT. However, recent studies in a number of fibrotic models have suggested that although EMT may not contribute directly to the fibroblast pool, it does augment fibrosis through secreted factors (Grande et al., 2015; Lovisa et al., 2015; Yao et al., 2018; Hill et al., 2019a; Hill et al., 2019b). Both EGFR (Burgel and Nadel, 2008; Cully and Downward, 2008; Vallath et al., 2014) and TGF-β have been demonstrated as key players in lung disease (Tatler and Jenkins, 2012; Saito et al., 2018).

TGF-β is responsible for a wide range of functions (Schuster and Krieglstein, 2002; Mukherjee et al., 2010; Carl et al., 2016; Cheng et al., 2016; Guo, 2017; Goldmann et al., 2018) and elucidating pathways which are altered in the lung upon its activation could be key in determining underlying mechanisms of lung disease. A number of studies have demonstrated TGF-β-induced EMT in the lung (Kasai et al., 2005; Chen et al., 2016). These studies have modeled EMT using A549 lung cells which harbour a *KRAS* mutation (Yoon et al., 2010), and also utilized a longer treatment time (Kasai et al., 2005; Chen et al., 2016). Primary ATII cells treated with TGF-β for 1 week were unable to induce EMT as assessed by reduction in E-cadherin, whereas treatment with EGF led to a reduction in E-cadherin protein levels suggesting induction of EMT (Yao et al., 2018). A recent microarray study also utilizing primary alveolar epithelial cells suggested EMT induction upon TGF-β treatment, however there was no reduction in traditional EMT markers E-cadherin or tight junctions (Goldmann et al., 2018). *KRAS* mutations occur in around 25–35% of cases of lung cancer (Kempf et al., 2016). Taking all these findings together we suggest that in a *KRAS*-driven environment, as is common in many cancers, EMT is induced more potently than by TGF-β. Here, we compare RAS-activated ATII^ER:KRASV12^ cells together with TGF-β treatment with TGF-β treatment alone; and it appears that EMT is induced to a greater extent than with TGF-β alone. ‘Hallmark EMT’ was identified as a top hit in the RAS-active proteomic analysis, and western blot analysis suggests that EMT was induced more potently than the TGF-β treated ATII^ER:KRASV12^ cells. Here we demonstrated in ATII cells that in this time period (24 h) there was no significant reduction in E-cadherin protein level. Using unbiased, quantitative proteomic analysis we demonstrated that TGF-β was unable to induce complete EMT in ATII cells in the given period and pathway analysis did not identify EMT as a pathway in ATII cells with TGF-β.

In comparison, RAS pathway activation was able to induce a hallmark EMT signature in ATII cells. In lung fibrosis, the EGFR pathway, upstream of RAS, has been implicated with a number of studies demonstrating mice expressing TGF-α develop lung fibrosis (Korfhagen et al., 1994; Hardie et al., 1997) and those lacking EGFR to be resistant to bleomycin induced fibrosis (Madtes et al., 1999). Clinically, IPF patients have been demonstrated to have EGFR mutations (Stella et al., 2014) or increased expression of EGFR (Tzouvelekis et al., 2013). We have previously demonstrated in ATII cells that EMT is induced via the EGFR-RAS-ERK pathway via ZEB1, and this mediated fibrosis via paracrine signaling (Yao et al., 2018). We propose that in the same manner that RAS-induced EMT in ATII ^ER:KRAS V12^ cells is via ZEB1, partial EMT is induced by TGF-β via Snail1 (Dang et al., 2011; Batlle et al., 2013; Li et al., 2014). As such, identifying the underlying mechanisms and more potential biomarkers/drug targets is clinically beneficial. Unbiased proteomic analysis of RAS-activated ATII cells identified a hallmark EMT signature, validated by protein and mRNA levels of a number of EMT markers. These findings are consistent with published literature on RAS-induced EMT in ATII cells (Yao et al., 2018).

Given the important roles of both RAS signaling and TGF-β in the pathogenesis of a number of lung diseases, including cancer and fibrosis, understanding the capacity of their signaling to induce different processes is particularly important (Kempf et al., 2016; Richeldi et al., 2017). Both have been proposed as potent inducers of EMT (Lamouille et al., 2014; Nieto et al., 2016), and it has been suggested in other contexts that when working together these can cooperate to have a synergistic effect through a crosstalk effect (Oft et al., 1996; Oft et al., 1998; Iglesias et al., 2000; Park et al., 2000; Gotzmann et al., 2002; Janda et al., 2002; Bates and Mercurio, 2003; Safina et al., 2009), and in the context of malignancy, RAS and TGF-β can act together to regulate epithelial cell plasticity (Janda et al., 2002). In a recent study in mammary epithelial cells, it was shown that when exogenous TGF-β was not present, inhibition of RAF/MEK/ERK was able to prevent EMT, but when exposed to TGF-β it is able to shortcut MEK to transition to mesenchymal cells (McFaline-Figueroa et al., 2019). An investigation into the transcriptomic response to TGF-β after 48 h found in human primary ATII cells both EMT and KRAS signaling to be induced (Goldmann et al., 2018). In ATII cells when RAS and TGF-β signaling are both induced, a hallmark EMT signature was identified and this was driven by RAS but a synergistic effect is not observed.

Taken together, these results indicate RAS activation to be a key inducer of EMT in the time period tested, whereas TGF-β does not seem to induce a complete EMT. When taken in the context of existing studies, it is clear that both time- and dose- of TGF-β treatment are particularly important when determining the effect on the EMT phenotype. Both TGF-β and RAS-activation are key signaling cascades in the pathogenesis of IPF, and despite studies in other contexts demonstrating a cooperation between these pathways, in ATII it appears RAS-activation drives EMT. While the exact details of these mechanism are yet to be fully elucidated in the context of IPF, understanding the interactions between these pathways will be helpful in the discovery of potential drug targets which may prevent the progression of fibrosis.

## Data Availability

The mass spectrometry proteomics data have been deposited to the ProteomeXchange Consortium via the PRIDE (Perez-Riverol et al., 2019) partner repository with the dataset identifier PXD023720.
