# Supplementary material for: Quantitative Proteomic Analysis in Alveolar Type II Cells Reveals the Different Capacities of RAS and TGF-β to Induce Epithelial–Mesenchymal Transition
Source: Front Mol Biosci. 2021 Mar 19;8:595712. doi: 10.3389/fmolb.2021.595712 (PMC8048883; doi:10.3389/fmolb.2021.595712)
Supplement: Supplementary file 1 [file datasheet1.docx]

Supplementary Material

# Supplementary Tables Legends

## Supplementary Table 1

Expression data for all proteins after limma processing between control and 4-OHT treatment (RAS-activated) in ATII ^ER:KRAS V12^ cells at 24 hrs, after being run through limma package (v. 3_34.2) in RStudio (v. 1.1.456).

## Supplementary Table 2

Hallmark EMT proteins significantly differentially expressed between control and 4-OHT treatment (RAS-activated) in ATII ^ER:KRAS V12^ cells at 24 hrs.

## Supplementary Table 3

Expression data for all proteins after limma processing between control and TGF-β treatment in ATII ^ER:KRAS V12^ cells at 24 hrs, after being run through limma package in RStudio.

## Supplementary Table 4

Hallmark EMT proteins significantly differentially expressed between control and TGF-β treatment in ATII ^ER:KRAS V12^ cells at 24 hrs.

## Supplementary Table 5

Expression data for all proteins after limma processing between control and TGF-β with 4-OHT (RAS-activated) treatment in ATII ^ER:KRAS V12^ cells at 24 hrs, after being run through limma package in RStudio.

## Supplementary Table 6

Hallmark EMT proteins significantly differentially expressed between control and TGF-β treatment with 4-OHT treatment (RAS-activated) in ATII ^ER:KRAS V12^ cells at 24 hrs.

# Supplementary Figures

## Supplementary Figure S1

Supplementary Figure 1 (a) Volcano plot comparing significantly (limma package; p value <0.05) changed proteins in ATII^ER:KRASV12^ cells treated with control and 250 nM 4-OHT (RAS-activated) treated for 24 hrs. Hallmark EMT proteins have been labelled. (b) Heatmap and hierarchal clustering analysis of imputed, normalized ATII^ER:KRASV12^ cells treated for 24 hrs with 250 nM 4-OHT compared with control.

## Supplementary Figure S2

Supplementary Figure 2 (a) Volcano plot comparing significantly (limma package; p value <0.05) changed proteins between control and 5ng/ml TGF-β treatment for 24 hrs in ATII^ER:KRASV12^ cells. Hallmark EMT proteins have been labelled. (b) Heatmap and hierarchal clustering analysis of imputed, normalized ATII^ER:KRASV12^ cells treated with 5ng/ml TGF-β for 24 hrs compared with control.

## Supplementary Figure S3

Supplementary Figure 3 Principal Component Analysis (PCA) of normalized proteome data from each sample. The first three principal components are shown in the plot and represent 23.1% of the variation (PC1), 14% of the variation (PC2) and 11.4% of the variation (PC3).

## Supplementary Figure S4

Supplementary Figure 4 The mRNA levels of *TGFBR1*, *TGFBR2*, and *TGFBR3* in ATII^ER:KRASV12^, normalized to *ACTB* (β-actin).

## Supplementary Figure S5

Supplementary Figure 5 (a) Protein levels of ZO1 in ATII^ER:KRASV12^ with indicated treatment from proteomics data. (b) Protein levels of ZO2 in ATII^ER:KRASV12^ with indicated treatment, from proteomics data. * P < 0.05. ** P < 0.01.
